# Cross-cultural adaptation of the Clear Communication Index to Brazilian Portuguese

**DOI:** 10.11606/s1518-8787.2020054001561

**Published:** 2020-03-09

**Authors:** Angélica Maria Cupertino Lopes Marinho, Cynthia Baur, Fernanda Morais Ferreira, Ana Cristina Borges-Oliveira, Mauro Henrique Nogueira Guimarães de Abreu

**Affiliations:** I Universidade Federal de Minas GeraisFaculdade de OdontologiaPrograma de Pós-graduação em OdontologiaBelo HorizonteMGBrasil Universidade Federal de Minas Gerais . Faculdade de Odontologia . Programa de Pós-graduação em Odontologia . Belo Horizonte , MG , Brasil; II University of MarylandSchool of Public HealthHerschel S. Horowitz Center for Health LiteracyMarylandEUA University of Maryland . School of Public Health . Herschel S. Horowitz Center for Health Literacy . College Park, Maryland , EUA; III Universidade Federal de Minas GeraisFaculdade de OdontologiaDepartamento de Saúde Bucal da Infância e AdolescênciaBelo HorizonteMGBrasil Universidade Federal de Minas Gerais . Faculdade de Odontologia . Departamento de Saúde Bucal da Infância e Adolescência . Belo Horizonte , MG , Brasil; IV Universidade Federal de Minas GeraisFaculdade de OdontologiaDepartamento de Odontologia Social e PreventivaBelo HorizonteMGBrasil Universidade Federal de Minas Gerais . Faculdade de Odontologia . Departamento de Odontologia Social e Preventiva . Belo Horizonte , MG , Brasil

**Keywords:** Health communication, Health education, Translations, Validation studies

## Abstract

**OBJECTIVE:**

To perform a cross-cultural adaptation of the Clear Communication Index instrument from the Centers for Disease Control and Prevention (CDC-CCI) from English to Brazilian Portuguese.

**METHODS:**

This study comprised initial discussion about the conceptual equivalence of the instrument by a committee formed by experts on health education. We performed translations, synthesis of translations, back-translations, revision by the committee, and linguistic revision. Semantic equivalence was obtained by analyzing the referential and general meaning of each item by the committee, resulting in a pre-final version of the instrument. Subsequently, thirty professionals with health sciences degrees performed a pre-test. These professionals used the pre-final version of the instrument to assess a health education material. A questionnaire was applied to evaluate the acceptability of the instrument, the understanding of each of the 20 items, as well as the individual and professional variables. We analyzed the scores attributed to the health education material, the variables related to healthcare professionals, the proportions of the acceptability of the instrument, and the comprehension of each item.

**RESULTS:**

After we obtained the conceptual equivalence of the instrument, the committee of experts, the instrument’s main author, and the linguist produced the pre-final version using two translations, a synthesis of the translations, and two back-translations. A general equivalence was maintained in 15 of the 20 items (75%), four of the items were slightly altered (20%), and one item was very altered (5%). Nineteen items presented referential equivalence or near equivalence (95%). We then carried out with the pre-test, in which the professionals used the pre-final version. Two items in the domains of “risks” and “main message” were unclear and needed to be revised.

**CONCLUSION:**

The process of cross-cultural adaptation of the Clear Communication Index provided an adapted version to the Brazilian Portuguese language.

## INTRODUCTION

Healthcare systems should pay attention to the quality of the messages offered to the public in printed and online materials ^1^ . Educational materials must be constructed based on an understanding of the target audience. Evaluations of educational materials, however, have shown several serious problems ^1 , 2 , 4^ . Hence, it is recommended that the evaluation and validation of health messages should occur prior to their availability to the public ^1 , 3 , 5^ .

The North American English language by Baur and Prue ^6^ validated in 2014 the Clear Communication Index from the U.S. Centers for Disease Control and Prevention (CDC-CCI) as one of the instruments available in the literature to evaluate information on health. The CDC-CCI is a tool comprised of a series of questions based on health literacy and communication research. Professionals can use the CDC-CCI to develop new messages and materials about health topics and to evaluate existing ones, regardless of the format or channel of distribution. The CDC’s communication researchers developed the CDC-CCI ^6^ to make sure the agency’s information is accurate, accessible, and actionable for its many different audiences. The instrument was also part of CDC’s implementation of the federal Plain Writing Act ^7^ , which requires federal government agencies to communicate clearly with the public, and the National Action Plan to Improve Health Literacy ^8^ . Both the law and action plan aim to establish clear and simple language for communication in health as the norm.

The CDC-CCI tool produces a numeric score that characterizes a message or material and is available in two versions. The full version consists of four open-ended introductory questions and 20 scored items that affect information clarity and audience comprehension, according to scientific literature ^6^ . The full version works best with longer-form print materials, such as handouts, flyers, webpages, and short reports. The modified Index has the same four open-ended introductory questions and only 13 of the 20 scored items. The modified Index can be used for short messages and materials, such as social media posts, infographics, and call center or podcast scripts. The 20 scored items have yes or no response options with an assigned point value. The scoring scale is 0-100 points with 90-100 points total as the recommended scoring range. The score represents how closely the material follows the Index criteria.

Although the CDC-CCI and similar tools were developed in English to evaluate English-language materials, there are few cross-culturally adapted tools for assessing health education materials in other languages and cultural contexts, such as Brazilian Portuguese. The development of better health education materials in the Brazilian Public Health System is of utmost importance for health promotion as well as to enhance access to information in health. We chose this index for four reasons. First, this instrument presents validity and reliability in its original version ^6^ . Second, it has the ability to assess “Main Message and Call to Action,” “Language,” “Information Design,” “State of the Science (scientific knowledge),” “Behavioral Recommendations,” “Numbers,” and “Risk in a short time,” ^8^ which is a necessary aspect in daily routines at healthcare services. Third, as it was developed to be used by healthcare professionals, mainly in public healthcare services, the index is a good fit for those responsible for health education and health publicity actions in Brazil. Fourth, since it was projected to be used during the creation and evaluation of health communication materials for a wide range of public and communication channels, it affects the general public. Thus, this study sought to conduct a cross-cultural adaptation of the original CDC-CCI instrument in English to the Brazilian Portuguese language (BR-CDC-CCI), evaluating the semantic and conceptual equivalence, acceptability, and comprehension of the items.

## METHODS

This study was approved by the Research Ethics Committee of the Universidade Federal de Minas Gerais (UFMG) under protocol CAAE 79108017.9.0000.5149.

We performed a cross-cultural adaptation of the CDC-CCI to help design and assess health messages and materials ^9 , 10^ . Four translators participated in the development of this research (two Brazilians and two Americans); a committee of experts consisting of Professors from the Schools of Dentistry and Pharmacy of the Universidade Federal de Minas Gerais (UFMG); a linguist; the main author of the original instrument, Cynthia Baur (CB), Professor at the University of Maryland, USA; and 30 primary healthcare professionals in public health, all volunteers, from a small city of the state of Minas Gerais, southeast Brazil. The sample size of 30 was similar to other studies in the cross-cultural adaptation of healthcare instruments ^11^ and consisted of a convenience sample of health professionals with higher education degrees who work at ten Primary Health Care Units in Minas Gerais. We approached these professionals at the Primary Health Care Unit where they work, and they provided written consent to participate in this project.

The CDC-CCI instrument, in its full version, consists of four introductory open-ended questions and 20 close-ended questions, with two answer options: “Yes” (score = 1) or “No” (score = 0), which the person who performs the scoring uses to evaluate the clarity and understanding of the information. The open-ended questions have no quantitative value, and each of the other 20 items is worth 1 point. Total scores vary from zero to 20 and are converted into a score on a scale of 0–100. The recommended minimum score is 90. The 20 questions encompass seven areas: “Main Message and Call to Action,” “Language,” “Information Design,” “State of the Science (scientific knowledge),” “Behavioral Recommendations,” “Numbers,” and “Risk.” ^6^ There is a short version called the “Modified Index,” with 13 questions in the same seven areas described above. For this study, we used the full version.

After consulting the author responsible for validating the instrument (CB), the cross-cultural adaptation process followed the recommendations set forth in international literature, which include conceptual and semantic equivalences ^9 , 10^ .

Conceptual equivalence refers to the validity of concepts (domains) explored in the instrument being adapted and is obtained through feedback from the group who will use the instrument; in this case, experts in health education. This equivalence establishes whether or not the instrument can be understood and accepted in the new cultural context. The conceptual equivalence establishes if the measurability of the seven domains in both the adapted instrument and the original are similar ^10^ .

Semantic equivalence depicts the correspondence of the meaning or correct translation of items (terms and words) ^9 , 10^ by a committee of experts. The semantic equivalence is based on the comparison of the meanings between the original instrument and the back-translations. Semantic equivalence can be evaluated from two aspects: a) the referential meaning that refers to similarities in meanings of items and can signal vocabulary or grammatical mistakes or discrepancies and b) the general meaning of each item that refers to the similarities of the ideas transmitted by the pairs of items.

**Figure 1:**
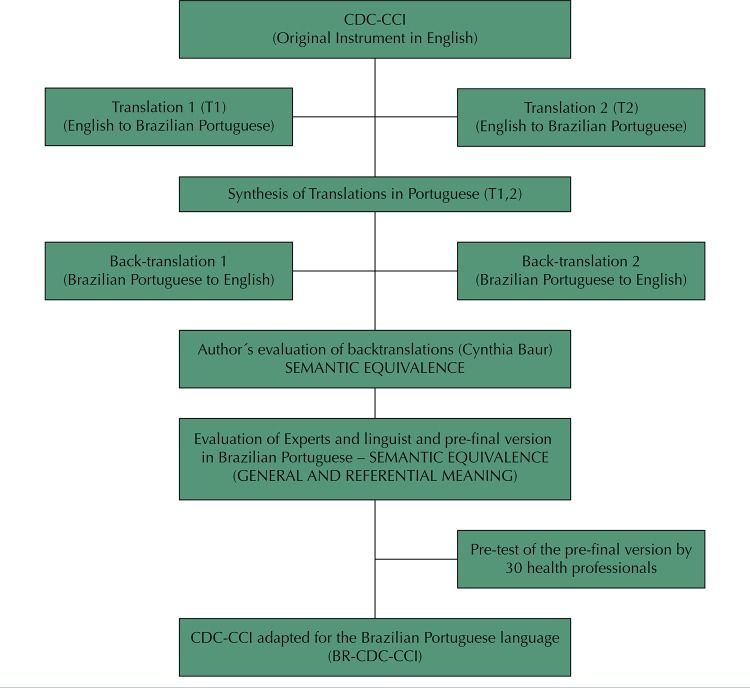
Flowchart of the steps of cross-cultural adaptation of the U.S. English language CDC-CCI to the Brazilian Portuguese language

The conceptual and semantic equivalence was obtained through translations, synthesis of the translations, back-translations, revision by a committee of experts in health, a linguist, and pre-tests ( Figure 1 ).

The committee of experts in health education assessed conceptual equivalence by checking the applicability, time of use, and capacity to measure (in its 20 questions in seven sections) the domain of “clarity of health materials” by the CDC-CCI in the Brazilian context. We discussed how different public and communication channels would use this instrument. In this moment, the researchers did not assess public health professionals.

Once the experts established conceptual equivalence, the subsequent step consisted of two translations from the original English to Brazilian Portuguese (T1 and T2), by two independent translators, both Brazilian with fluency in English. One of the translators had knowledge and practice in health, having familiarity with the terms and concepts present in the instrument. The other had no specific knowledge regarding the instrument’s technical terms.

Four researchers, experts in health sciences, compared the two translations (T1 and T2), identifying discrepancies. This comparison generated a synthesis of the translations (T1 and T2), which aimed to identify possible difficulties in understanding the instrument. They compared the meaning of words in the different languages (English and Brazilian Portuguese) so that the same results were obtained in both translations.

Following the synthesis of the two translations (T1 and T2), two separate translators, native speakers from the United States with fluency in Brazilian Portuguese, performed independently two back-translations to English. The back-translators had no knowledge of the objectives of this work and did not have access to the original instrument. We sent the back-translations to the main author responsible for the validation of the original instrument (CB) in order to evaluate the quality of the translations and suggest modifications in the instrument. After this stage, the first version of the instrument was completed.

Subsequently, we conducted a review of the back-translations and a synthesis of the translations. Thus, a committee of experts composed of the same four researchers in health sciences, all four translators, a linguist, and two health professionals with experience in health research took the instrument in the original version as a reference. The establishment of a committee of experts was necessary for the achievement of a consensus regarding the conceptual and semantic equivalence of the items.

The committee of experts received the back-translation and original version of the CDC-CCI. For referential meaning, the committee evaluated these two versions without knowing which was the original and which had been back-translated ^11 , 14^ . A visual analog scale was used for referential meaning evaluation. The committee judged the equivalence of the pairs of statements (original and back-translated) by consensus, with a scale from zero to 100% using the following categories: “non-equivalent” (< 80%), “near equivalent” (80−89%), and “equivalent” (90−100%).

**Figure 2:**
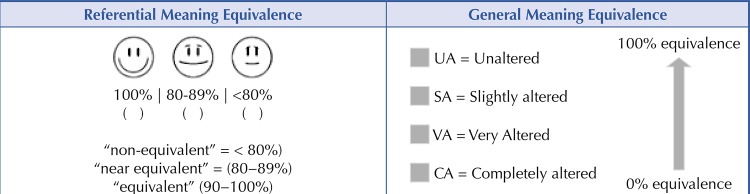
The scales used in the semantic equivalence of Br-CDC-CCI. Belo Horizonte, 2019

For the general meaning evaluation, the committee used a scale from zero to 100%. They evaluated each pair of statements, having to reach a consensus. They classified them as: unaltered (UA), slightly altered (SA), very altered (VA), and completely altered (CA) ^10 , 11 , 14 , 15^ . In this step, the committee was aware of the two versions (the original and the back-translated). The scales used for the semantic equivalence can be seen in Figure 2 .

Following semantic equivalence (referential and general meaning evaluation), the research team approached primary healthcare professionals at their places of work in Minas Gerais to perform a pre-test of the Brazilian Portuguese draft instrument (BR-CDC-CCI) in May and June 2018. The researchers asked the healthcare professionals to use the instrument to assess the health education material “Rational Use of Drugs” ^16^ from the Ministry of Health. The research team chose this health education material because it is publicly available, about a common topic, and includes features that the CDC-CCI is designed to evaluate. The professionals used the draft BR-CDC-CCI instrument to score the “Rational Use of Drugs” material. Each of the 30 professionals completed questionnaires on the acceptability of the instrument as a whole, the understanding of each of the 20 items, and individual and professional profiles. For acceptability, we included a general and dichotomous question (“Yes” or “No”): “Do you think that this instrument would be acceptable for Brazilian professionals?” Regarding the understanding of each item, we included the following question: “After you read the BR-CDC-CCI items and evaluation criteria, mark those that were understood (“Yes”) and those that were not understood (“No”). For the items not understood, write in the corresponding space any problem with comprehension you had.” Professionals also reported the amount of time spent answering the items of the instrument. In addition, the professionals answered questions about their age, sex, time since graduation, type of health sciences degree, public service time, and whether they had completed a graduate degree.

We analyzed the scores from the 30 primary care professionals using the Statistical Package for Social Sciences (SPSS for Windows, version 25.0, SPSS Inc., Chicago, IL). The variables related to the profiles and individual characteristics of the healthcare professionals were statistically analyzed by measuring the frequency and central tendency. The statistical analyses also included the calculation of the proportions of acceptability of the instrument and comprehension of each item from the BR-CDC-CCI.

## RESULTS

We carried out the cross-cultural adaptation systematically. The first assessment by the experts showed that the applicability, time of use, and capacity to obtain the necessary domains of “clarity of health materials” could be obtained in the Brazilian version of the CDC-CCI. The group considered that the instrument would be used for healthcare professionals when creating and evaluating health information materials in a wide range of communication channels and for a diverse public. They considered the instrument necessary, practical, and useful in the Brazilian context and approved its Conceptual Equivalence.

We changed some sections of the English CDC-CCI in which cities, units of measurement, or U.S. public institutions were mentioned. Examples of this first step were changes of “ *Springfield* ” to “São Paulo” (item 18), “ *ounces* ” to “grams” (item 16), and “ *Public health organizations (...) (ASTHO)* ” to Brazilian Health Organizations such as “CONASS, CONASEMS.” Other considerations included the exclusion of links to U.S. government agencies and institutions, where we inserted Brazilian equivalents. The author responsible for the validation of the original instrument (CB) also highlighted the need to maintain the term “primary audience” instead of “main audience” throughout the instrument. The idea of “primary” is for educators to focus on the most important audience who will use the information, even though other “secondary” audiences may also see the information if it is posted on a website, for example.

Table shows the BR-CDC-CCI adaptation process, from the synthesis of translations to the adapted version, including the conceptual and semantic equivalence evaluations. For referential equivalence, nineteen items presented equivalence or near equivalence (95%), while only question 18 was “non-equivalent.” In the general equivalence between the original instrument and the back-translation, 15 of the 20 items were unaltered (UA=75%); four of the items, questions 2, 3, 5 and 18, were slightly altered (SA=20%); and one item, question 1, was very altered (VA=5%).

**Table:** Semantic Equivalence (Referential Meaning Equivalence—RME and General Meaning Equivalence—GME) between the original CDC-CCI (in English) and the version in Brazilian Portuguese by a committee of experts, and Conceptual Equivalence (Comprehension-Compr. and Acceptability-Accept.) of the pre-final Brazilian version of the CDC-CCI by health professionals.

Baur & Prue	Brazilian translator 1 and 2 + Commitee	(American translator 1) + Baur revision	(American translator 2) + Baur revision	Semantic Equivalence (Committee)		Conceptual equivalence (Health Professionals)		Adapted Version of the BR-CDC-CCI
Original	Synthesis of translations	Backtranslation 1	Backtranslation 2	RME	GME	Compr (n=30)	Accept (n=30)	
**1. Does the material have a main message?** The main message is the one thing you want to communicate to a person or group and what they should remember. A topic, such as heart disease or seasonal flu, is not a main message. If the material contains multiple messages and no main message, answer no. NOTE: If you answered **No** for Question 1, **check 0 for Question 2-4** and **go on** to Question 5.	**1. O material contém uma mensagem principal?** Uma mensagem principal é a única coisa que você quer comunicar a uma pessoa ou grupo e que eles devem se lembrar. Um tópico, tal como doença cardíaca ou gripe sazonal, não é uma mensagem principal. Se o material contiver várias mensagens e nenhuma mensagem principal, responda Não. Se você respondeu **Não** para a Pergunta 1 **, marque 0 para as Perguntas 2 a 4 e siga** para a Pergunta 5.	**1. Does the material contain a main message?** A main message is the only thing that you want to communicate to a person or group and that they should remember. One topic, such as heart disease and seasonal flu, is not a main message. If the material contains many messages and no main message, answer no. NOTE: If you answered **No** to question 1, **check 0 for Question 2-4** and **skip** to Question 5.	**1. Does the material contain one main message?** A main message is the one thing you want to communicate to a person or group that they must remember. A topic, such as heart disease or seasonal flu, is not a main message statement. If the material contains several messages and no main message, answer no. NOTE: If you answered **No** to Question 1, **score 0 for Questions 2-4** and **continue** to Question 5.	**70%**	VA	Yes = 25 (83%) No = 05 (17%)	100%	**1. O material contém uma mensagem principal?** Uma mensagem principal é a única coisa que você quer comunicar a uma pessoa ou grupo e que eles devem se lembrar. Não se considera mensagem principal a apresentação de apenas um tópico, tal como “doença cardíaca” ou “gripe sazonal”. Se o material contiver várias mensagens e nenhuma mensagem principal, responda não. **NOTA:** Se você respondeu **Não** para a questão **1** , marque **0 para a questão 2-4** e **siga** para a questão **5** .
**2. Is the main message at the top, the beginning, or in the front of the material?** The main message should be in the first paragraph or section. A section is a block of text between headers. For a Web material, the first section must be fully visible without scrolling.	**2. A mensagem principal está no topo, no início ou na parte da frente do material?** A mensagem principal deve estar no primeiro parágrafo ou seção. Uma seção é um bloco de texto entre cabeçalhos. Para materiais da web, a primeira sessão deve estar completamente visível sem rolagem.	**2. Is the main message at the top, in the beginning, or on the front part of the material?** The main message should be in the first paragraph or section. A section is a block of text between the headers. For a Web material, the first section must be fully visible without scrolling.	**2. Is the main message at the top, beginning, or front of the material?** The main message must be in the first paragraph or section. A section is a block of text between headings. For a Web material, the first section must be fully visible without scrolling.	**80%**	SA	Yes = 30 (100%) No = 0 (0%)	100%	**2. A mensagem principal está no topo, no início ou na parte da frente do material?** A mensagem principal deve estar no primeiro parágrafo ou seção. Uma seção é um bloco de texto entre cabeçalhos. Para materiais da Internet, a mensagem principal deve estar na primeira página.
**3. Is the main message highlighted with visual cues?** If the main message is highlighted through the use of fonts, colors, shapes, lines, arrows, or headings, such as “What you need to know,” answer yes.	**3. A mensagem principal é enfatizada com indicações visuais?** Se a mensagem principal for enfatizada com fonte, cor, formas, linhas, setas ou títulos, tais como “O que você precisa saber”, responder sim	**3. Is the main message emphasized with visual prompts?** If the main message is emphasized with font, color, shapes, lines, arrows, or titles, such as “What you need to know,” answer yes.	**3. Is the main message emphasized with visual cues?** If the main message is emphasized with font, color, shapes, lines, arrows or headings, such as “What you need to know,” answer yes.	**100%**	SA	Yes = 30 (100%) No = 0 (0%)	100%	**3. A mensagem principal é enfatizada com indicações visuais?** Se a mensagem principal for enfatizada com fonte, cor, formas, linhas, setas ou títulos, tais como “O que você precisa saber”, responda sim.
**4. Does the material contain at least one visual element that conveys or supports the main message?** Consider photographs, drawings, graphics, and infographics as visual elements. If the visual element does not have a caption or labels, answer “No.” If there are human figures that are not performing the recommended behaviors, answer “No.”	**4. O material contém pelo menos um elemento visual que transmita ou dê suporte à mensagem principal?** Considere como exemplos de elementos visuais fotografias, desenhos, gráficos e infográficos. Se o elemento visual não tiver uma legenda ou rótulos, responder “Não”. Se tiver figuras humanas que não estejam realizando os comportamentos recomendados, responder “Não”.	**4. Does the material contain at least one visual element that transmits or gives support to the main message?** Consider as examples of visual elements photographs, designs, graphs, and infographics. If the visual element does not have a legend or label, answer no. If it has human figures that are not performing the recommended behaviors, answer no.	**04. Does the material contain at least one visual that conveys or supports the main message?** For example, count photographs, line drawings, graphs and infographics as visuals. If the visual does not have a caption or labels, answer no. If the visual has human figures who are not performing the recommended behaviors, answer no.	**100%**	UA	Yes = 30 (100%) No = 0 (0%)	100%	**4. O material contém pelo menos um elemento visual que transmita ou dê suporte à mensagem principal?** Considere como exemplos de elementos visuais: fotografias, desenhos, gráficos e infográficos. Se o elemento visual não tiver uma legenda ou rótulos, responda não. Se tiver figuras humanas que não estejam realizando os comportamentos recomendados, responda não.
**5. Does the material include one or more calls to action for the primary audience?** If the material includes a specific behavioral recommendation, a stimulus for more information, a request to share information with another person, or a broad call for program or policy change, answer yes. If the call to action is for someone other than the primary audience, answer no.	**5. O material inclui uma ou mais chamadas para a ação para o** **público-alvo** **?** Se o material incluir uma recomendação comportamental específica, um estímulo para obter mais informações, um pedido para compartilhar informações com outra pessoa, ou uma ampla chamada para mudança de programa ou política , responda sim. Se a chamada para a ação for para alguém que não seja o público-alvo , responda não.	**5. Does the material include one or more calls to action for the main audience?** If the material includes a specific behavioral recommendation, a stimulus to obtain more information, a request to share information with another person, or a broad call to change the program or policy, answer Yes. If the call to action is for someone that is not from the main audience, answer No.	**5. Does the material include one or more calls to action for the primary audience?** If the material includes a specific behavioral recommendation, a prompt to get more information, a request to share information with someone else, or a broad call for program or policy change, answer yes. If the call to action is for someone other than the primary audience, answer no.	**90%**	SA	Yes = 29 (97%) No =1 (3%)	100%	**5. O material inclui uma ou mais chamadas para ação direcionadas ao** **público?** Se o material incluir a recomendação de um comportamento específico, um estímulo para obter mais informações, um pedido para compartilhar informações com outra pessoa, ou uma ampla chamada para mudança de programa de saúde , responda sim. Se a chamada para ação for para alguém que não seja o público, responda não
**6. Is the main message and call to action used in the active voice?** If only the main message or only the call to action use the active voice, answer no. If you answered no to Questions 1 or 5, answer no.	**6. A mensagem principal e a chamada para ação usam a voz ativa?** Se apenas a mensagem principal ou apenas a chamada para ação usar a voz ativa, responda não. Se você respondeu não às perguntas 1 ou 5, responda não.	**6. Do the main message and call to action use the active voice?** If only the main message or only the call to action uses the active voice, answer No. If you answered No to questions 1 or 3, answer No.	**6. Do both the main message and the call to action use the active voice?** If only the main message or only the call to action uses the active voice, answer no. If you answered no to #1 or #5, answer no.	**100%**	UA	Yes = 30 (100%) No = 0 (0%)	100%	**6. A mensagem principal e a chamada para ação usam a voz ativa?** Se apenas a mensagem principal ou apenas a chamada para ação usam a voz ativa, responda não. Se você respondeu não às questões 1 ou 5, marque não.
**7. Does the material always use words that the primary audience uses?** If all specialized or unknown terms are explained or described (not only defined) the first time they are used, answer yes. Acronyms and abbreviations should be written in full and explained, if unknown to the public.	**7. O material usa sempre palavras que o público-alvo** **utiliza** **?** Se todos os termos especializados ou desconhecidos são explicados ou descritos (não apenas definidos) na primeira vez em que são usados, responda sim. Siglas e abreviaturas devem ser escritas por extenso e explicadas, se desconhecidas para o público-alvo.	**7. Does the material always use words that the main target audience uses?** If all of the specialized or unknown terms are explained or described (not only defined) in their first usage, answer Yes. Acronyms and abbreviations should be written out and explained if unknown to the audience.	**07. Does the material always use language the primary audience would use?** If all specialized or unfamiliar terms are explained or described (not just defined) the first time they are used, answer yes. Acronyms and abbreviations must be spelled out and explained if unfamiliar to the audience.	**100%**	UA	Yes = 30 (100%) No = 0 (0%)	100%	**7. O material sempre usa palavras que o público** **está acostumado?** Se todos os termos especializados ou desconhecidos forem explicados ou descritos (não apenas definidos) na primeira vez em que são usados, responda sim. Siglas e abreviaturas devem ser escritas por extenso e explicadas, caso sejam desconhecidas pelo público.
**8. Does the material use lists with bullets or numbers?** If the material has a list of more than 7 items, and the list isn’t broken down into sub-lists, answer “No.” If the list consists only of additional information or references, or is placed at the end of the material, answer “No.”	**8. O material usa listas com marcadores ou números?** Se o material contiver uma lista com mais de 7 itens, e a lista não for dividida em sub-listas, responder “Não”. Se a lista for apenas de informações adicionais ou de referências, ou estiver no final do material, responder “Não”.	**8. Does the material use lists with markers or numbers?** If the material contains a list with more than 7 items, and the list is not divided into sub-lists, answer no. If the list is only of additional information or references, or if it is at the end of the material, answer no.	**8. Does the material use bulleted or numbered lists?** If the material contains a list with more than 7 items, and the list is not broken up into sub-lists, answer no. If the list is for additional information or references only or at the end of the material, answer no.	**100%**	UA	Yes = 30 (100%) No=0 (0%)	100%	**8. O material usa listas com marcadores ou números?** Se o material abranger uma lista com mais de sete itens, e a lista não for dividida em sublistas, responda não. Se a lista for apenas de informações adicionais ou de referências, ou estiver no final do material, responda não.
**9. Is the material organized in blocks with headings?** This item applies to texts and lists. If the blocks contain more than one idea each, answer “No.” If the headings do not correspond to the blocks of information, answer “No.”	**9. O material é organizado em blocos com títulos?** Este item aplica-se a textos e listas. Se os blocos contiverem mais de uma ideia cada, responder “Não”. Se os títulos não estiverem de acordo com os blocos de informação, responder “Não”.	**9. Is the material organized in blocks with titles?** This item is applicable to texts and lists. If the blocks contain more than one idea each, answer no. If the titles are not in accordance with the blocks of information, answer no.	**9. Is the material organized in chunks with headings?** This item applies to prose text and lists. If the chunks contain more than one idea each, answer no. If the headings do not match the information chunks, answer no.	**100%**	UA	Yes = 30 (100%) No = 0 (0%)	100%	**9. O material é organizado em blocos com títulos?** Este item aplica-se a textos e listas. Se os blocos contiverem mais de uma ideia cada, responda não. Se os títulos não estiverem de acordo com os blocos de informação, responda não
**10. Is the most important information the primary audience needs to take away summarized in the first paragraph or section?** The most important information should include the main message. A section is a block of text between headers. For a Web material, the first section must be fully visible without scrolling.	**10. A informação mais importante que o público-alvo principal precisa está resumida no primeiro parágrafo ou seção?** A informação mais importante deve incluir a mensagem principal. Uma seção é um bloco de texto entre cabeçalhos. Para um material da web, a primeira sessão deve ser completamente visível sem rolagem da pagina .	**10. Is the most important information that the main target audience needs summarized in the first paragraph or section?** The most important information should include the main message. One section is a block of text between the headers. (For a Web material, the first section must be fully visible without scrolling.	**10. Is the most important information the primary audience needs summarized in the first paragraph or section?** The most important information must include the main message. A section is a block of text between headings. For a Web material, the first section must be fully visible without scrolling.	**90%**	UA	Yes = 30 (100%) No = 0 (0%)	100%	**10. A informação mais importante para o público l encontra-se resumida no primeiro parágrafo ou seção?** A informação mais importante deve incluir a mensagem principal. Uma seção é um bloco de texto entre cabeçalhos. Para um material da Internet, a primeira seção deve ser totalmente visível na primeira página.
**11. Does the material explain which trusted sources, such as experts and government representatives, know and do not know about the subject matter?** If the material addresses both, answer “Yes.” If the material addresses only one (what is known or what is not known), answer no.	**11. O material explica o que fontes fidedignas, tais como especialistas no assunto e os representantes governamentais, sabem e não sabem sobre o assunto?** Se o material abordar os dois, responda “Sim”. Se o material abordar apenas um (o que se sabe ou o que não se sabe), responder não.	**11. Does the material explain which reliable sources, such as specialists on the issue and governmental representatives, know or do not know about the issue?** If the material addresses both, answer yes. If the material addresses only one (what one knows or does not know), answer no.	**11. Does the material explain what authoritative sources, such as subject matter experts and agency spokespersons, know and do not know about the topic?** If the material addresses both, answer yes. If the material addresses only one (what is known or not known), answer no.	**100%**	UA	Yes = 30 (100%) No = 0 (0%)	100%	**11. O material explica o que fontes confiáveis, tais como especialistas no assunto e representantes governamentais, sabem e não sabem sobre o tema?** Se o material abordar os dois, responda sim. Se o material abordar apenas um (o que se sabe ou não se sabe), responda não.
**12. Does the material include one or more behavioral recommendations for the primary audience?** If no, STOP here and do not answer Part B.	**12. O material inclui uma ou mais recomendações comportamentais para o público-alvo?** Se não, PARE aqui e não responda a Parte B.	**12. Does the material include one or more behavioral recommendations for the main audience?** If not, STOP here and do not answer Part B.	**12. Does the material include one or more behavioral recommendations for the primary audience?** If no, STOP here and do not score Part B.	**100%**	UA	Yes = 30 (100%) No = 0 (0%)	100%	**12. O material inclui uma ou mais recomendações de comportamento para o público principal?** Se não, PARE aqui e não marque a Parte B.
**13. Does the material explain why the behavioral recommendation(s) is (are) important for the primary audience?** If the material uses only numbers to explain the importance of behavioral recommendation without offering other relevant information to the audience, answer no.	**13. O material explica por que a(s) recomendação(ões) comportamental(is) é(são) importante(s) para o público-alvo?** Se o material usa apenas números para explicar a importância da recomendação comportamental sem outras informações relevantes para o público-alvo, responda não.	**13. Does the material explain why the behavioral recommendation(s) is important for the main audience?** If the material has only numbers to explain the importance of the behavioral recommendation without other relevant information for the public, answer No.	**13. Does the material explain why the behavioral recommendation(s) is important?** If you offer only numbers to explain the importance of the behavioral recommendation with no other relevant information for the audience, answer no.	**100%**	UA	Yes = 30 (100%) No = 0 (0%)	100%	**13. O material explica por que a(s) recomendação(s) de comportamento(s) são importantes para o público principal?** Se você oferecer apenas números para explicar a importância da recomendação de comportamento sem outras informações relevantes para o público, responda não.
**14. Do the behavioral recommendations include specific instructions on how to carry out the behavior?** This may include step-by-step instructions or a simple description (for example: Look for cereals that have 100% of the recommended daily amount of folic acid). If the material includes information about when and how to get in touch with a physician or other healthcare professional, answer “Yes.” If the material mentions when and how often to carry out a behavior, answer “Yes.”	**14. As recomendações comportamentais incluem instruções específicas sobre como realizar o comportamento?** Isso pode incluir instruções passo-a-passo ou uma descrição simples (por exemplo: Procure cereais com 100% de valor diário de ácido fólico). Se o material incluir informações sobre quando e como entrar em contato com um médico ou outro profissional de saúde, responder “Sim”. Se o material mencionar quando e com que frequência realizar um comportamento, responder “Sim”.	**14. Do the behavioral recommendations include specific instructions about how to perform the behavior?** This may include step-by-step instructions or a simple description (for example: Look for cereals with 100% of the daily value of folic acid). If the material includes information about when and how to get in contact with a doctor or other healthcare professional, answer yes. If the material mentions when and how often to perform a behavior, answer yes.	**14. Does the behavioral recommendation(s) include specific directions about how to perform the behavior?** This may include step-by-step directions or a simple description (for example: Look for cereal with 100% daily value of folic acid). If the material includes information about when or how to contact a medical provider or health official, answer yes. If the material mentions when or how often to perform a behavior, answer yes.	**90%**	UA	Yes = 29 (97%) No = 1 (3%)	100%	**14. As recomendações de comportamento incluem instruções específicas sobre como realizar o comportamento?** Isso pode incluir instruções passo-a-passo ou uma descrição simples (por exemplo: Procure cereais com 100% de valor diário de ácido fólico). Se o material incluir informações sobre quando e como entrar em contato com um médico ou outro profissional de saúde, responda sim. Se o material mencionar quando e com que frequência realizar um comportamento, responda sim
**15. Does the material always have numbers that the primary audience uses?** Many people find numbers distracting or confusing. Make sure the numbers in the material are familiar and necessary to base or explain the main message. Otherwise, take them out. Whole numbers are used by most people. The types of numbers used vary for each audience.	**15. O material sempre apresenta números que o público-alvo principal utiliza?** Muitas pessoas acham que números distraem ou confundem. Certifique-se de que os números no material sejam familiares e necessários para embasar ou explicar a mensagem principal. Caso contrário, exclua os números. Números inteiros são usados pela maioria das pessoas. Os tipos de números utilizados variam para cada público.	**15. Does the material always present numbers that the main target audience uses?** Many people think that numbers distract or confuse. Make sure that the numbers in the material are familiar and necessary to support or explain the main message. If not, exclude the numbers. Whole numbers are used by most people. The types of numbers used vary for each audience.	**15. Does the material always present numbers the primary audience uses?** Many audiences find numbers distracting or confusing. Make sure the numbers in the material are both familiar and necessary to support or explain the main message statement. If not, delete them. Whole numbers are used by most audiences. The types of numbers used will vary for each audience.	**100%**	UA	Yes = 30 (100%) No = 0 (0%)	100%	**15. O material sempre apresenta números que o público utiliza?** Muitas pessoas acham que números distraem ou confundem. Certifique-se de que os números no material sejam familiares e necessários para embasar ou explicar a mensagem principal. Caso contrário, exclua os números. Números inteiros são usados pela maioria das pessoas. Os tipos de números utilizados variam para cada público.
**16. Does the material always explain what the numbers mean?** For example, “the recommended amount of meat as part of a healthy meal is 3 to 4 ounces, which is similar to the size of a playing card.”	**16. O material sempre explica o que os números significam?** Por exemplo, “a quantidade de carne recomendada como parte de uma refeição saudável é de 3 a 4 gramas – o que é semelhante ao tamanho de uma carta de baralho”.	**16. Does the material always explain what the numbers mean?** For example, “the quantity of meat recommended as part of a healthy meal is from 3 to 4 ounces—which is similar to the size of a playing card.”	**16. Does the material always explain what the numbers mean?** For example, “The amount of meat recommended as part of a healthy meal is 3 to 4 ounces—it will look about the same size as a deck of cards.”	**100%**	UA	Yes = 30 (100%) No = 0 (0%)	100%	**16. O material sempre explica o que os números significam?** Por exemplo, “a quantidade de carne recomendada como parte de uma refeição saudável é de 85 a 113 gramas – o que é semelhante ao tamanho de uma carta de baralho”.
**17. Should the audience perform mathematical calculations?** Addition, subtraction, multiplication, and division involve calculations. The calculation of a common denominator for comparison purposes is a mathematical calculation. Use the same denominator, even for absolute risk (e.g., 1 of 3), on all material so the primary audience does not need to calculate. NOTE: **for this item, Yes is scored as 0 and No is scored as 1.**	**17. O público-alvo deverá realizar cálculos matemáticos?** Adição, subtração, multiplicação e divisão envolvem cálculos. O cálculo de um denominador comum para fins de comparação é um cálculo matemático. Use o mesmo denominador, mesmo para risco absoluto (exemplo: 1 de 3), em todo o material para que o público-alvo não precise calcular. **NOTA: para este item, Sim é pontuado 0 e Não é pontuado 1.**	**17. Should the audience do mathematical calculations?** Addition, subtraction, multiplication, and division involve calculations. The calculation of a common denominator for comparison is a mathematical calculation. Use the same denominator, even for absolute risk (example: 1 of 3), in all material so that the audience does not need to calculate. NOTE: **for this item, Yes is scored 0 and No is scored 1.**	**17. Does the audience have to conduct mathematical calculations?** Adding, subtracting, multiplying, and dividing involve calculations. Calculating a common denominator for the purposes of comparison is a mathematical calculation. Use the same denominator, even for absolute risk (example: 1 out of 3), throughout the material so that audiences do not have to calculate. NOTE: **for this item, Yes is scored 0 and No is scored 1.**	**100%**	UA	Yes = 30 (100%) No = 0 (0%)	100%	**17. O público deverá realizar cálculos matemáticos?** Adicionar, subtrair, multiplicar e dividir envolvem cálculos. O cálculo de um denominador comum para fins de comparação é um cálculo matemático. Use o mesmo denominador, mesmo para risco absoluto (exemplo: 1 de 3), em todo o material para que o público não precise calcular. **NOTA** : **para este item, o “sim” corresponde a 0 e o “não” corresponde a 1.**
**18. Does the material explain the nature of the risk?** If the material identifies the threat or possible harm and how and why people may be affected by it, answer yes. If the material only mentions the threat or possible harm without any explanation, answer no. For example, in saying that there are 1,000 new cases of a contagious disease in Springfield, does the material also state that people in Springfield may be more likely to contract the disease? Why could they be more prone to becoming ill and how serious is the threat of the disease?	**18. O material explica a natureza do risco?** Se o material indicar a ameaça ou dano e como e porque as pessoas podem ser afetadas, responda sim. Se o material tiver apenas a ameaça ou o dano, mas nenhuma explicação, responda não. Por exemplo, ao afirmar que existem 1000 novos casos de uma doença contagiosa em São Paulo, o material afirma também que as pessoas em São Paulo podem ser mais propensas a contrair a doença? Por que elas podem ser mais propensas a adoecer e quão séria é a ameaça da doença?	**18. Does the material explain the nature of the risk** ? If the material indicates the threat or harm and how and why people can be affected, answer Yes. If the material has only the threat or the harm, but no explanation, answer No. For example, when affirming that there are 1,000 new cases of a contagious disease in Springfield, does the material also affirm that the people in Springfield may be more prone to contracting the disease? Why might they be more prone to getting sick and how serious is the threat of disease?	**18. Does the material explain the nature of the risk?** If the material states the threat or harm and how and why people may be affected, answer yes. If the material has only the threat or harm but no explanation, answer no. For example, if the material states there are 1,000 new cases of a contagious disease in Springfield, does it also state that people in Springfield may be more likely to get the disease, why may they be more likely, and how serious the threat of the disease is?	**90%**	SA	Yes = 24 (80%) No = 6 (20%)	100%	**18. O material explica a natureza do risco?** Se o material apresenta o risco, o modo e o motivo pelo qual as pessoas podem ser afetadas, responda sim. Se o material apresenta apenas o risco, mas nenhuma explicação, responda não. Por exemplo, ao apresentar a ocorrência de mil novos casos de uma doença contagiosa em São Paulo, o material também declara que as pessoas em São Paulo podem estar mais propensas a contrair a doença, o motivo para tal e quão sério é o risco?
**19. Does the material address both the risks and the benefits of the recommended behaviors?** This includes real risks and benefits and those perceived by your audience. If the material addresses only risks or only benefits, answer no. If no behavioral recommendation is presented, the answer does not apply (NA).	**19. O material aborda tanto os riscos quanto os benefícios dos comportamentos recomendados?** Isso inclui riscos e benefícios reais e aqueles percebidos pelo seu público. Se o material abordar apenas riscos ou apenas benefícios, responda não. Se nenhuma recomendação comportamental for apresentada, responda não se aplica (NA).	**19. Does the material address both the risks and the benefits of recommended behaviors?** This includes real risks and benefits and those perceived by their audience. If the material addresses only risks or only benefits, answer No. If no behavioral recommendation is presented, answer Not Applicable (NA).	**19. Does the material address both the risks and benefits of the recommended behaviors?** This includes actual risks and benefits and those perceived by your audience. If the material addresses only risks or only benefits, answer no. If no behavioral recommendation is presented, answer not applicable (NA).	**100%**	UA	Yes = 30 (100%) No = 0 (0%) NA= 0 (0%)	100%	**19. O material aborda tanto os riscos quanto os benefícios dos comportamentos recomendados?** Isso inclui riscos e benefícios reais e aqueles percebidos pelo seu público. Se o material abordar apenas riscos ou apenas benefícios, responda não. Se nenhuma recomendação de comportamento foi apresentada, responda não se aplica (NA).
**20. If the material uses numerical probability to describe the risk, is the probability also explained by words or visuals?** Examples of probability information in a risk message are numbers (such as 1 in 5 or 20%). If the material presents numerical risk and also uses text to explain the probability, answer yes. If the material presents numerical risk and also uses a visual resource to explain the probability, answer yes. If the material presents only numerical risk, answer no. If the material does not include this type of probability information, answer does not apply (NA).	**20. Se o material usa a probabilidade numérica para descrever o risco, a probabilidade também é explicada com palavras ou com recursos visuais?** Exemplos de informações de probabilidade em uma mensagem de risco são números (como 1 em 5 ou 20%). Se o material apresenta risco numérico e também usa texto para explicar a probabilidade, responda sim. Se o material apresenta risco numérico e também usa um recurso visual para explicar a probabilidade, responda sim. Se o material apresentar apenas risco numérico, responda não. Se o material não incluir esse tipo de informação de probabilidade, responda não se aplica (NA).	**20. If the material uses the numerical probability to describe the risk, is the probability also explained with words or with visual aids?** Examples of probability information in a risk message are numbers (such as 1 in 5 or 20%). If the material presents a numerical risk and also uses text to explain the probability, answer Yes. If the material presents numeric risk and also uses visual resource to explain the probability, answer yes If the material presents only a numerical risk, answer No. If the material does not include this type of probability information, answer Not Applicable (NA).	**20. If the material uses numeric probability to describe risk, is the probability also explained with words or a visual resource?** Examples of probability information in a risk message are numbers (such as 1 in 5 or 20%). If the material presents numeric risk and also uses text to explain the probability, answer yes. If the material presents numeric risk and also uses a visual resource to explain the probability, answer yes. If the material only presents numeric risk, answer no. If the material does not include this type of probability information, answer not applicable (NA).	**90%**	UA	Yes = 30 (100%) No = 0 (0%)	100%	**20. A probabilidade numérica usada para descrever o risco também é explicada com palavras ou recursos visuais?** Números são exemplos de informações de probabilidade em uma mensagem de risco (tais como 1 em 5 ou 20%). Se o material apresenta risco numérico e também usa texto para explicar a probabilidade, responda sim. Se o material apresenta risco numérico e também usa um recurso visual para explicar a probabilidade, responda sim. Se o material apresenta apenas risco numérico, responda não. Se o material não inclui esse tipo de informação de probabilidade, responda não se aplica (NA).

The committee of experts highlighted the need for a review by a linguist, who evaluated the material and made recommendations. The main changes occurred in questions 18, considered no referential equivalent, and 1, which was very altered. The changes, as suggested by consensus, are presented in Table . In accordance with the assessment of the committee, question 1 presented a misunderstanding by not specifying what the “Main Message” would be in the context of the information, and for not defining what the educational materials would be. The committee observed linguistic and grammatical errors in questions 2, 3, 5, and 18 and did not approve them as maximum equivalence. Among these were the differences between the terms “section” and “session” in question 2; “highlighted” and “emphasized” in question 3; “calls to action for the primary audience” and “calls to action directed to the public” in question 5; and “web” and “internet” in question 18. We corrected the differences with the aid of a linguist.

The majority of the primary healthcare professionals involved in the pre-test were female (87%), with an average age of 36.8 years (range 24–49), average time since completion of undergraduate degree of 13 years (range 3–31), and 53% with a graduate-level degree. Most of the professionals were nurses (57%), 20% were dentists, and 23% were healthcare professionals in other areas (Speech Therapist, Physician, Nutritionist, and Psychologist). In relation to professionals’ understanding of the BR-CDC-CCI items, six professionals (20%) did not understand question 18 regarding the nature of the risk, and five (17%) did not understand question 1 regarding the main message. One (3%) professional did not understand question 5, regarding call to action, and question 14, regarding behavioral directions. All 30 professionals understood the rest of the questions ( Table ).

In view of these considerations, the committee and the linguist re-evaluated questions 1, 5, 14, and 18. Items 5 and 14 were considered confusing by one professional each, since they did not understand the meaning of the words “primary audience” and the example of the nutrition behavioral recommendation using folic acid. As items 1 and 18 continued to be a problem, the committee observed that the pre-final version should be clarified, and certain sentences shortened. The committee changed the order of some words and removed others, making the final Brazilian version clearer than the previous one ( Table ).

## DISCUSSION

The CDC-CCI instrument aims to contribute to the improved performance of healthcare professionals who create educational materials, since it leads them to critically analyze their own communicative capacity ^17 , 18^ . The use of the CDC-CCI can improve the development and transmission of health messages, as well as the public’s orientation regarding actions and better results in health ^19^ .

Low health literacy is considered a social determinant of health, with low literacy being a predictor of worse health outcomes ^20 , 21^ . Professionals’ use of tools to adapt health messages and materials for low literacy audiences can contribute to improvements in the public’s adherence to care and therapeutic outcomes, as well as to reduce social inequalities ^22^ . The BR-CDC-CCI, after its final validation, can offer healthcare professionals a practical resource, guiding them in the creation and evaluation of materials and educational messages in health, following the example of other studies in the literature ^18 , 19 , 23^ .

The adaptation of the CDC-CCI instrument is a crucial stage, since it provides an opportunity to test its feasibility in Brazil. This study obtained the results of the conceptual and semantic equivalence using robust methods that were used in other processes for adapting instruments from English to Brazilian Portuguese ^11^ . One adapted instrument must be equivalent to the source instrument in such a way that its meaning is the same for the majority of the desired population ^10^ in their different cultural and linguistic contexts. For this, original instruments and adaptation “must dialogue with each other” according to a team of judges. These judges should have the ability to understand whether or not the representation of the original instrument is similar to the representation in its final population, which, in this context, is a final population of healthcare professionals or others involved in the development of health education materials.

In this research, two items showed that divergences need to be rigorously analyzed, and the corrected versions should be included in the final format of an instrument. The misunderstanding generated by items 1 (main message) and 18 (nature of risk) made it difficult to evaluate the domains “Main Message” and “Risk” in the Brazilian context. These items were misunderstood in the process of obtaining equivalence (referential and general) and remained critical during the pre-test with 30 health professionals. Such misunderstanding may have two possible causes: semantic/syntax difficulty or the professionals did not consider these two items relevant for evaluation. The second reason could result in the exclusion of items for the Brazilian context, given that the “Main Message” and “Risk” domains would perform differently than the original instrument. As the problems detected were of syntax, our corrections allowed the two domains to follow the original instrument.

Following the evaluation by the committee, the pre-test version of question 1 remained the same as in the synthesis of the translations, but the pre-test with the 30 professionals showed they were confused about what the “Main Message” would be. Modifications to the order of words made the question clearer. In question 18, the word “damage,” contained in the explanation of the question, was replaced by “risk,” repeating the term already used in the question to reinforce the meaning in the original version of the CDC-CCI. We corrected the misunderstanding on what the instrument calls the “Main Message” and “Risk” during the adaptation process. These findings reinforce those found in other studies, highlighting the importance of the work of the committee of experts and pre-test in cross-cultural adaptations ^11 , 12 , 14 , 15^ .

This study also observed the importance of the participation of translators compatible with the criteria that the literature advocates. The independent translations and back-translations allowed us to locate the errors and discrepancies in ambiguous or unmatched items between the two languages. Translation by both a translator with health training and one without this training made it possible to detect a greater range of difficulties in understanding the instrument ^11^ . The author of the instrument observed additional discrepancies or misalignments not detected during translation, synthesis, or back-translations, giving greater credibility and fidelity to the initial proposal.

A future study will evaluate some psychometric properties of the BR-CDC-CCI, after we complete this cross-cultural adaptation. The adapted instrument still needs to undergo a process of evaluation in larger groups of professionals and materials. Despite the fact that the Brazilian National Health System has the same principles –such as comprehensiveness, universality, equitability– for the whole country, some cultural differences between regions and professional groups are likely to occur. Testing the BR-CDC-CCI with a larger number of health professionals located in different Brazilian regions and professionals with different training and experiences is necessary. These evaluation methods could allow the assessment of reliability and validity. We also will do a qualitative assessment to compare this instrument with others ^6 , 17 , 18^ . Other instruments that evaluate the quality of written clinical treatment choices could also be used when we evaluate and create health education materials ^24 , 25^ . In conclusion, the process of cross-cultural adaptation of the Clear Communication Index provided an adapted instrument to the Brazilian Portuguese language, which this is the first step in a longer process of testing and refining the BR-CDC-CCI for broad use among health professionals.
